# Longitudinal study of quality of life in advanced cancer patients on home parenteral nutrition

**DOI:** 10.1002/cam4.1111

**Published:** 2017-05-29

**Authors:** Paolo Cotogni, Luca De Carli, Roberto Passera, Maria Luisa Amerio, Elena Agnello, Maurizio Fadda, Marta Ossola, Taira Monge, Antonella De Francesco, Federico Bozzetti

**Affiliations:** ^1^ Department of Anesthesia and Intensive Care University of Turin Turin Italy; ^2^ Unit of Parenteral Nutrition in Oncology S. Giovanni Battista Hospital Turin Italy; ^3^ Clinical Nutrition S. Giovanni Battista Hospital Turin Italy; ^4^ Nuclear Medicine Division S. Giovanni Battista Hospital Turin Italy; ^5^ Clinical Nutrition Cardinal Massaia Hospital Asti Italy; ^6^ Faculty of Medicine University of Milan Milan Italy

**Keywords:** EORTC QLQ‐C30 questionnaire, home care, nutrition, oncologic treatment, palliative care, quality of life, supportive care

## Abstract

Since there is little knowledge regarding the quality of life (QoL) of cancer patients on home parenteral nutrition (HPN), we planned a prospective, longitudinal, double‐center study to investigate the changes of QoL in these patients. One hundred and eleven adult cancer patients who were candidates for HPN following the indications of the European guidelines were consecutively enrolled. For QoL analysis, EORTC QLQ‐C30 questionnaires were filled at the HPN start and after 1, 2, 3, and 4 months, and scores changes over time were analyzed according to the univariate mixed‐effects linear model for repeated measures. Most patients had gastrointestinal cancers, were severely malnourished, and were in stage IV; two‐thirds were still receiving oncologic treatments. Median weight loss over 3 months and body mass index were 11.7% and 20.7, respectively. Median survival was 4.7 (1–42) months; 67 and 34% of patients survived 3 and 6 months, respectively. Global QoL, physical functioning, role functioning, emotional functioning, appetite loss, and fatigue scores had a statistically significant trend over time (*P *<* *0.001, *P *<* *0.001, *P *=* *0.007, *P *<* *0.001, *P *=* *0.004, *P *=* *0.022, respectively). At the univariate analyses, the determinants significantly associated with changes in trend over time for physical, role, and emotional functioning were oncologic treatments (*P *<* *0.001, *P *=* *0.014, *P *=* *0.040, respectively) and for appetite loss they were weight loss and Karnofsky performance status (*P *=* *0.003, *P *=* *0.023, respectively). Global QoL, physical, role, and emotional functioning improved during HPN even in advanced cancer patients on oncologic treatments.

## Introduction

Recent studies 1, 2 have shown that home parenteral nutrition (HPN) might prolong survival in incurable cancer patients unable to eat, beyond the usually expected time span. Prognostic determinants have been defined 1 and their power was further validated in a large series of patients 2.

There is, however, little information about their quality of life (QoL), although in the common perspective of patients, relatives, and caregivers, the shorter is the life expectancy the more relevant the purpose of maintaining an acceptable QoL. A survey 3 on the relevance of QoL for advanced cancer patients showed that only 22% would choose palliative chemotherapy, in preference to supportive care alone, to benefit from the associated 3‐month additional survival advantage; conversely, most would choose chemotherapy if it substantially reduced adverse symptoms even without prolonging life. Although QoL represents a crucial issue in these patients, few prospective studies have so far focused on this topic 4, 5, 6 and investigated patients on HPN with or without concurrent oncologic treatments.

Thus, we planned a study to analyze the QoL in advanced cancer patients on HPN and to investigate whether the combination with oncologic treatments correlates with changes of QoL.

## Materials and Methods

### Study design

This is a prospective, longitudinal, observational, double‐center study carried out from October 1, 2011 through September 30, 2013 in a 1200‐bed tertiary referral care, university hospital and a 551‐bed tertiary care hospital. The Ethics Committee approved the study protocol and a written informed consent was obtained from each patient.

All adult cancer patients who were candidates for HPN according to the European guidelines 7, 8 were consecutively enrolled when meeting all the following criteria: proven and prolonged failure to meet nutrition requirements by the oral or enteral route, with impending risk of death due to malnutrition; life expectancy >2 months; Karnofsky performance status (KPS) >50; control of pain; absence of severe organ dysfunctions; written informed consent confirming that the patient accepted this modality of nutrition support; approval by the physician responsible for HPN, the oncologist and the general practitioner; presence of environmental conditions compatible with HPN; availability of an in‐home caregiver; and availability of a specifically trained nursing team dedicated to the patient home care, as provided by the Public Health Service.

Patients were closely monitored by the physician responsible for HPN through regularly scheduled and structured telephone interviews (at least every 15 days) and home visits by nursing team and general practitioner (initially daily for 2–3 weeks and at least every 7 days thereafter). After adequate training, their caregivers administered the HPN bag. HPN was delivered 10‐ to 14‐hour per day night‐time using standard industrially assembled ‘all‐in‐one’ bags. Every 30 days from the HPN start (range ±5 days), an outpatient re‐evaluation by both the physician and the dietitian was performed.

All patients were followed up until withdrawal of HPN. HPN was withdrawn because of worsening clinical state (e.g., uncontrolled or refractory symptoms, progression of the tumor spread or onset of major organ failure), death or when oral adequate nutrition by oral route had been restored (i.e., a food intake above 75% of normal requirements in the preceding week).

Diagnosis and management of infectious complications closely followed the guidelines 9.

### QoL analysis

For QoL analysis, the European Organization for Research and Treatment of Cancer Quality of Life Questionnaire Core 30 (EORTC QLQ‐C30) Version 3.0 was used; in particular, the version validated in Italian language 10. It is a 30‐item cancer‐specific questionnaire that consists of: five functioning scales (physical, role, cognitive, emotional, and social); eight symptom scales (fatigue, nausea/vomiting, pain, dyspnea, sleep disturbance, appetite loss, constipation, and diarrhea); financial well‐being scale (financial impact); and a global scale (global QoL) 11, 12.

The questionnaire was filled out by the patients at the HPN start in an outpatient setting, in the presence of a physician or dietitian responsible for HPN in case of need of assistance. Subsequently, it was filled out by the patients themselves during outpatient re‐evaluations after 1, 2, 3, and 4 months.

Data presented as T0, T1, T2, T3, and T4 are referred to the date of the HPN start and 1, 2, 3, and 4 subsequent months. The raw scores were linearly transformed to give standard scores in the range of 0 to 100 for each of the functioning and symptom scales. Higher scores in the global and functioning scales indicate better QoL, while lower scores in the symptom scales indicate better QoL.

### Statistical analysis

The mixed‐effects linear model for repeated measures represents a proper statistical method to assess possible changes in QoL scores over time both within and among patients, allowing for differing numbers of measures *per* patient and accounting for missing values, by incorporation of all available data into a single model spanning the entire follow‐up period. These characteristics make this model ideal for investigating the QoL changes over time. The primary outcomes were independent trends over time of the EORTC QLQ‐C30 scales and their potential modifications by different determinants. All these time series were longitudinally measured at four time‐points (1, 2, 3, and 4 months) after the HPN start; these repeated measures were used as a dependent variables in univariate mixed‐effects linear models. Since the distribution of any time series by single time‐point was Gaussian, all models were estimated without log‐transforming their values. The univariate analyses were performed for the following independent covariates: age (>60 vs. ≤60 years), gender (female vs. male), weight loss (WL) (≥10% vs. <10%), KPS, tumor site (other locations vs. upper gastrointestinal tract), stage (IV vs. III), oncologic treatments (any vs. none), metastasis (any vs. none), and patient‐generated subjective global assessment (PG‐SGA) score (C vs. B). Overall survival was calculated as the number of months between the date of the HPN start and that of patient death from any cause, with censoring at the date of last follow‐up assessment in live subjects.

All results for continuous variables are expressed as the mean (standard deviation). All reported *P*‐values were obtained by the two‐sided exact method, at the conventional 5% significance level. Data were analyzed by R 3.2.3 software (R Foundation for Statistical Computing, Vienna, Austria).

## Results

### Patients

One hundred‐eleven adult cancer patients were consecutively enrolled in this study and followed till death or weaning from HPN for a total of 17,542 HPN‐days. No patient was lost to follow‐up. Table 1 shows the main characteristics of patients. Our population included all patients with advanced disease. Most patients had a gastrointestinal cancer, were severely malnourished, and were (sub)obstructed; 65% were still receiving oncologic treatments (chemotherapy 61, radiation therapy 2 and both treatments 9 patients, respectively). All patients had a residual ‐but insufficient‐ oral food intake (a median of 500 Kcal). Thus, supplemental HPN provided a median amount of 1000–1250 kcal *per* day, considering a need of 20–25 kcal/kg/day for bedridden or 25–30 kcal/kg/day for ambulatory patients and 1–1.5 g/kg/day amino acids. HPN was withdrawn in most cases because of worsening clinical state or death. In 27 patients (24%) HPN was withdrawn because adequate nutrition intake by the oral route have been restored: 18 of 72 (25%) were on oncologic treatments and 9 of 39 (23%) were without oncologic treatments. The incidence of catheter‐related bloodstream infections was 0.33 *per* 1000 catheter‐days.

**Table 1:** Patients’ characteristics

	All patients	Treatment^a^	No treatment
*N* (%)	111	72 (65)	39 (35)
Female gender, *n* (%)	54 (49)	36 (50)	18 (46)
Age (years), median (range)	62 (32–79)	59 (32–78)	66 (44–79)
Tumor site, *n* (%)			
Stomach	38 (34)	31 (43)	7 (18)
Colon/rectum	21 (19)	15 (21)	6 (15)
Pancreas/biliary system	20 (18)	13 (18)	7 (18)
Esophagus	10 (9)	4 (6)	6 (15)
Lung	10 (9)	3 (4)	7 (18)
Ovary	2 (2)	1 (1)	1 (3)
Others	10 (9)	5 (7)	5 (13)
Stage, *n* (%)			
III	25 (23)	15 (21)	10 (26)
IV	86 (77)	57 (79)	29 (74)
Metastasis, *n* (%)	73 (68)	48 (67)	25 (64)
Karnofsky PS, median (range)	70 (60–80)	70 (60–80)	70 (60–80)
BMI, median (range)	20.7 (13.5–29.5)	21.2 (13.5–29.5)	19.7 (13.5–28.7)
Weight loss^b^, (%), median (range)	11.7 (0–38.3)	10.6 (2.1–38.3)	12.3 (0–31.7)
PG‐SGA category, *n* (%)			
B	41 (37)	29 (40)	12 (31)
C	70 (63)	43 (60)	27 (69)
Oral food intake (Kcal), median (range)	500 (200–1300)	500 (200–1250)	550 (200–1300)
Indication for HPN, *n* (%)			
Intestinal sub(obstruction)^c^	90 (81)	59 (82)	31 (80)
SBS; high‐output ileostomy or fistula	14 (13)	8 (11)	6 (15)
EN not tolerated or feasible	7 (6)	5 (7)	2 (5)
HPN duration (days), median (range)	137 (21–576)	139 (27–576)	86 (21–317)

PS, performance status; BMI, body mass index; PG‐SGA, patient‐generated subjective global assessment; SBS, short bowel syndrome; EN, enteral nutrition; HPN, home parenteral nutrition.

a Treatment: chemotherapy and/or radiation therapy.

b In the last 3 months before HPN.

c Intra‐abdominal recurrence and/or peritoneal carcinomatosis.

**Figure 1:**
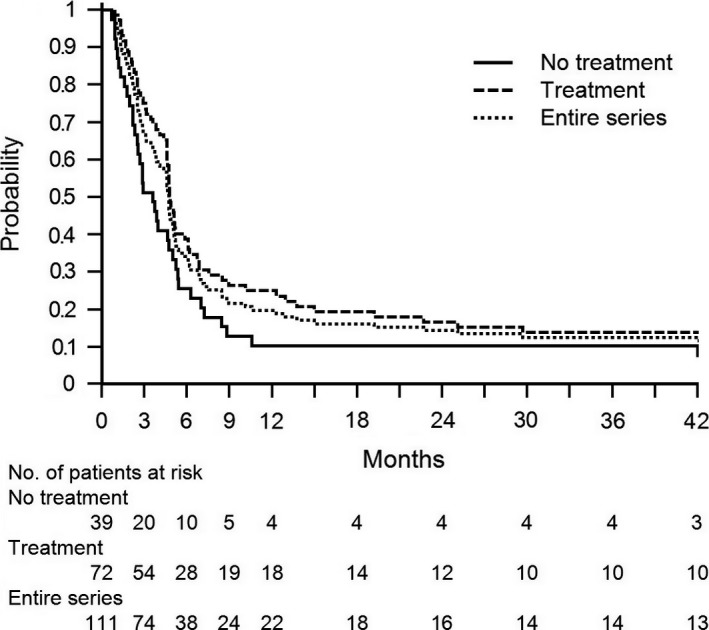
Overall survival curves. Kaplan–Meier overall survival curves of the entire series, treated, and nontreated patients.

Forty‐seven patients (42%) died during the study period of QoL analysis, 24 of 72 (33%) were on oncologic treatments and 23 of 39 (59%) were without oncologic treatments. Overall survival of the entire series was 67% at 3 months and 34% at 6 months. At the time of this analysis (June 2015), 98 (88%) patients died while 13 were still alive (median overall survival: 4.7 months, range 1–42) (Fig. 1). No HPN‐related mortality occurred.

### QoL analysis

**Figure 2:**
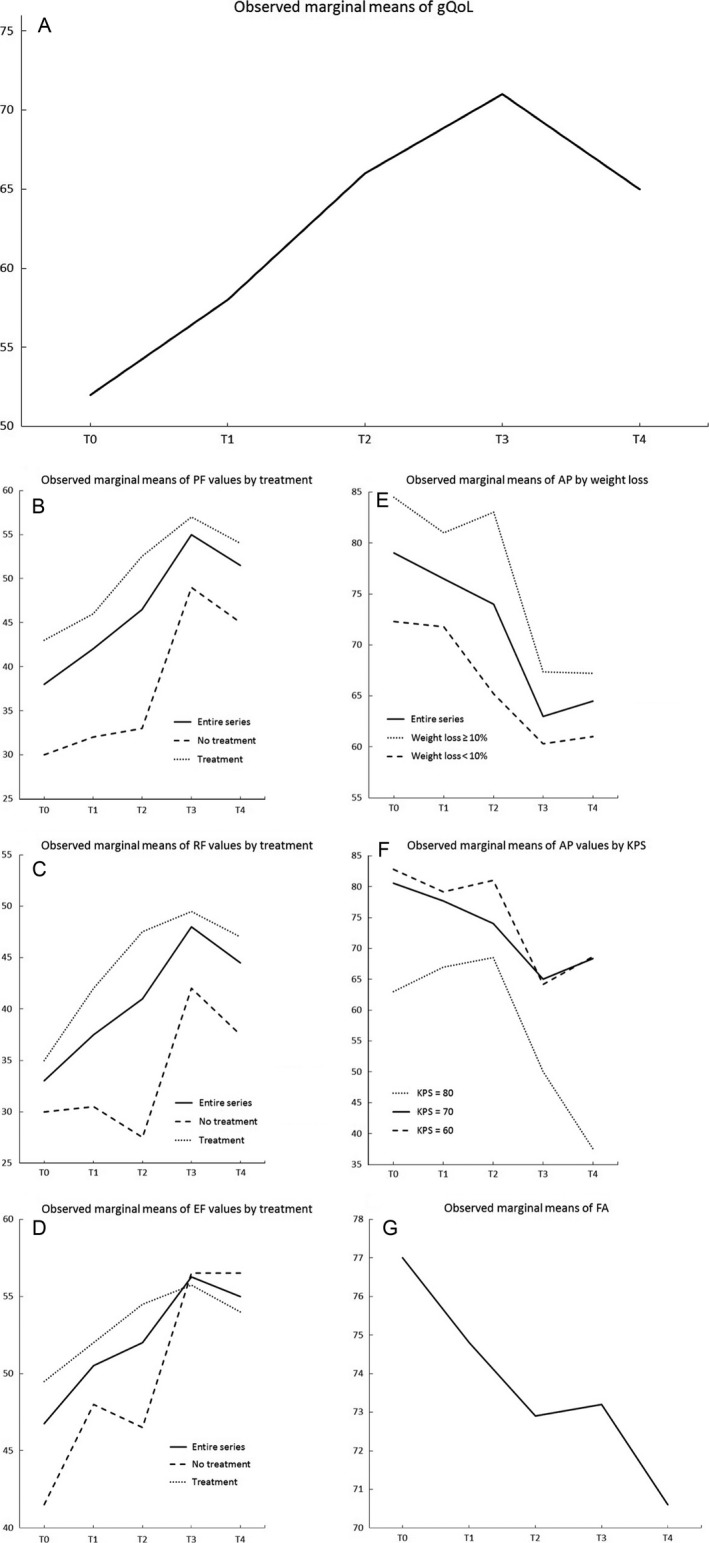
Patients’ scores for the EORTC QLQ‐C30 scales at different time‐points. Scores (range from 0 to 100) are depicted, as observed marginal mean by single time‐point, for the independent time series that had a statistically significant trend over time at univariate mixed‐effects linear models for repeated measures. Determinants significantly associated at the univariate analyses with changes in trend over time are showed. In figures A, B, C and D higher scores indicating better QoL. In figures E, F, and G lower scores indicating better QoL. T0, at the HPN start; T1, after 1 month; T2, after 2 months; T3, after 3 months; T4, after 4 months; EORTC QLQ‐C30, European Organization for Research and Treatment of Cancer Quality of Life Questionnaire‐Core 30; gQoL, global Quality of Life; PF, physical functioning; RF, role functioning; EF, emotional functioning; AP, appetite loss; KPS, Karnofsky performance status; FA, fatigue.

The questionnaire was well accepted by the patients. On average, it required less than 15 min to be completed and was filled out by themselves with no assistance. Patients’ scores for the EORTC QLQ‐C30 scales at different time‐points are depicted in Table 2. The univariate mixed‐effects linear model for repeated measures of EORTC QLQ‐C30 scales (Table 3) demonstrated that global QoL, physical functioning, role functioning, emotional functioning, appetite loss and fatigue scores had a statistically significant trend over time (*P *<* *0.001, *P *<* *0.001, *P *=* *0.007, *P *<* *0.001, *P *=* *0.004, *P *=* *0.022, respectively) (Fig. 2). At the univariate analyses, the determinants significantly associated with changes in trend over time for physical, role, and emotional functioning were treatments (*P *<* *0.001, *P *=* *0.014, *P *=* *0.040, respectively) and for appetite loss they were WL and KPS (*P *=* *0.003, *P *=* *0.023, respectively) (Table 3).

**Table 2:** Patients’ scores for the EORTC QLQ‐C30 scales at different time‐points

Time‐points	T0	T1	T2	T3	T4
Number of available measures	111/111 (100%)	97/111 (87.4%)	76/111 (68.5%)	54/111 (48.6%)	49/111 (44.1%)
Global QoL^a^	52 (17)	58 (17)	66 (17)	71 (14)	66 (16)
PF^a^	38 (22)	42 (22)	46 (21)	55 (16)	52 (17)
RF^a^	33 (24)	38 (26)	41 (24)	48 (19)	45 (20)
EF^a^	47 (16)	51 (17)	52 (13)	56 (12)	55 (12)
CF^a^	58 (17)	59 (18)	62 (16)	62 (17)	63 (12)
SF^a^	53 (21)	54 (20)	56 (21)	60 (16)	57 (21)
AP^b^	79 (26)	77 (23)	74 (22)	63 (26)	64 (24)
FA^b^	77 (17)	75 (16)	73 (17)	73 (18)	71 (16)
NV^b^	56 (25)	52 (20)	54 (20)	54 (18)	54 (20)
FI^b^	36 (21)	36 (19)	36 (19)	34 (15)	35 (16)

Scores are indicated as mean (standard deviation) by single time‐point for all the independent time series.

T0, at the start of HPN; T1, after 1 month; T2, after 2 months; T3, after 3 months; T4, after 4 months; EORTC QLQ‐C30, European Organization for Research and Treatment of Cancer Quality of Life (QoL) Questionnaire‐Core; PF, physical functioning; RF, role functioning; EF, emotional functioning; CF, cognitive functioning; SF, social functioning; FA, fatigue; AP, appetite loss; NV, nausea and vomiting; FI, financial impact.

a Scores range from 0 to 100, with higher scores indicating better QoL.

b Scores range from 0 to 100, with lower scores indicating better QoL.

**Table 3:** Determinants for trend over time for the EORTC QLQ‐C30 scales

	GlobalQoL	PF	RF	EF	CF	SF	AP	FA	NV	FI
Trend over time	**<0.001**	**<0.001**	**0.007**	**<0.001**	0.619	0.202	**0.004**	**0.022**	0.366	0.455
Determinants										
Gender	0.879	0.903	0.993	0.452	0.871	0.617	0.682	0.448	0.086	0.447
Age	0.479	0.863	0.264	0.931	0.153	0.143	0.202	0.665	0.949	0.002
Weight loss	0.247	0.106	0.289	0.300	0.996	0.746	**0.003**	0.936	0.257	0.206
Karnofsky PS	0.055	0.086	0.120	0.106	0.317	0.677	**0.023**	0.711	0.712	0.266
Tumour site	0.419	0.845	0.521	0.298	0.534	0.385	0.161	0.067	0.890	0.133
Stage	0.712	0.167	0.314	0.648	0.618	0.512	0.877	0.439	0.916	0.520
Treatment^a^	0.252	**<0.001**	**0.014**	**0.040**	0.040	0.087	0.162	0.680	0.934	0.264
Metastasis	0.694	0.985	0.809	0.811	0.264	0.728	0.281	0.098	0.536	0.721
PG‐SGA	0.279	0.739	0.909	0.710	0.367	0.613	0.192	0.809	0.228	0.363

Statistical analysis was performed using univariate mixed‐effects linear models for repeated measures; *P*‐values for any single univariate model.

EORTC QLQ‐C30, European Organization for Research and Treatment of Cancer Quality of Life (QoL) Questionnaire‐Core 30; PF, physical functioning; RF, role functioning; EF, emotional functioning; CF, cognitive functioning; SF, social functioning; FA, fatigue; AP, appetite loss; NV, nausea and vomiting; FI, financial impact; PS, performance status; PG‐SGA, patient‐generated subjective global assessment.

a Chemotherapy and/or radiation therapy.

The *P*‐values < 0.05 are indicated in bold.

## Discussion

This study showed that patients with advanced malignancy requiring a nutritional supplementation through HPN maintained their QoL or even showed an improvement in some scores according to the EORTC QLQ‐C30. The items which significantly improved were the domains of global QoL, physical, role, and emotional functioning, as well as appetite loss and fatigue.

It is noteworthy that improvements in physical, role, and emotional functioning were present in patients receiving a concurrent oncologic treatment as chemotherapy, radiation therapy or both. This raises the issue about the true determinants of benefit when the global care is represented by a multi‐component approach which includes nutritional support, oncologic treatment, and a general intensive assistance as in this experience.

The quality of care in this case series was high, as reflected by the low catheter infection rate achieved through a strict protocol 13. Indeed, as it was assessed by a group of experts, the catheter care is an important outcome indicator in patients on HPN 14.

In our study, we observed that patients receiving oncologic treatments showed higher QoL scores than patients with no treatment. This finding may reflect the better clinical conditions of patients undergoing an oncologic therapy. In fact at the HPN start, physical and emotional functioning scores were significantly higher in treated versus nontreated patients (data not shown). However, the relative increase of QoL scores in patients on oncologic therapy was less relevant than in untreated patients. Recently, a large study showed that palliative chemotherapy, despite its widespread use in patients with end‐stage cancer, did not improve QoL for patients with moderate or poor performance status and actually worsened QoL for patients with good performance status 15. Therefore, it could be possible that in our patients the benefits of HPN outweighed the adverse effects of the oncologic treatments.

Four months after the HPN start, we observed a decline of global QoL in our cancer patients. This finding is due to the fact that the patients were nearing the end of their lives (median survival was 4.7 months); however, this decline is not statistically significant.

The anxiety that surrounds eating and the distress that it causes to patients and their families, as well as the consequent escalation into arguments over food, determines negative repercussions for all of them. These drawbacks are well known in the everyday practice and are described in the literature regarding malnourished/hypophagic patients 16. Conversely, Overall et al 17 reported the sense of relief and security of both patients and relatives when the nutritional needs were met through HPN. Therefore, it is likely that the combination of nutritional support and intensive home care of these patients may have played a role in improving global QoL and, mainly, emotional functioning.

In our study, the 24% of patients were able to be weaned‐off HPN for regaining oral autonomy. The mechanisms through which HPN may restore an adequate nutrition intake by the oral route were not clearly defined. It is reasonable that this improvement is not primarily achieved by a single intervention (i.e., HPN), but by both the patient's ability to cope with the disease itself and the efficacy of different concomitant medical treatments.

Regarding the specific impact of HPN, we supposed that the following elements played a role in the ability to regain oral food intake in our patient population. First, in this study, patients were not aphagic; conversely, all patients had a residual ‐but insufficient‐ oral food intake. Second, these patients showed significant improvements in appetite loss as well as in physical functioning and fatigue. As a matter of fact, analysis of determinants of QoL showed that appetite loss was correlated with higher WL and lower KPS. This was not an unexpected finding since the impact that a poor nutrient intake has on WL and performance status is well known in literature and has been confirmed in a large prospective study 18. Finally, some patients during HPN reported the ability to resume some activities of daily living (e.g., eating meals, taking care of family, social activities, yard work, straightening up the house, and so forth). According to the literature 19, in these patients, HPN was able to postpone loss of autonomy allowing them to maintain for a longer period these activities of daily living.

The improvements in physical and role functioning found in our study are in agreement with the results of Lundholm et al. 20 who reported that cancer patients receiving a nutritional support had prolonged survival associated to improved energy balance and a greater maximum exercise capacity. Similarly, Pelzer et al. 21 showed that supplemental HPN for about 4 months was able to improve the phase angle and to get at least a temporary benefit or stabilization of the nutritional status in the majority of advanced pancreatic cancer patients.

Our findings are also in agreement with the recent study by Vashi et al 5. who described in 52 cancer patients a significant improvement in QoL after 1, 2, and 3 months. Similarly, Culine et al 6 reported after 28 days of HPN a significant increase in QoL in 437 cancer patients. However, it is noteworthy that in both these studies all patients were under oncologic therapy and about one‐third was in stage I or II, that is with a cancer burden probably lower than in our series. Our study further differentiates from the Vashi 5 and Culine 6 studies for the higher number of patients enrolled and the greater number of repeated QoL evaluations, respectively.

Our study presents some limitations, the most important being that we could not explore the relation between tumor response and QoL in patients receiving an oncologic treatment. Even if we speculate that the efficacy of chemo/radiation therapy may play some role in gaining a better state of health in advanced cancer patients, this issue would have required a joint effort of oncologists and nutritionists, an approach never achieved so far even in other prestigious cancer centers. A second limitation is the lack of a control group. However, the indication for HPN in these patients is based on a condition of severe hypophagia or its permissive effect for further oncologic treatments in malnourished patients. Both these conditions would not ethically allow a randomization for a no‐HPN arm. Finally, the statistical analysis used is sophisticated and well suited and applicable for missing values appearing in a random fashion. However, missing data are successively accumulating as the study advances and missing values will not be randomly distributed but could be worse than the values of the subjects remaining on study. The points of strength are the prospective design together with an overzealous care of the patients which has allowed to follow up all of them till death or weaning from HPN and to maintain a good quality of home care.

In conclusion, it would appear that our experience has an intermediate place among studies on QoL in cancer patients on HPN. Specifically, at one extreme there is the study of Bozzetti et al 4. which reported stable QoL indexes till 2–3 months before death in incurable aphagic patients on total HPN, and on the other the Vashi and Culine studies 5, 6 which considered a supplemental HPN in potentially curable patients. In fact, our study included cancer patients in stage III and IV, most receiving a supplemental HPN and being on cancer treatment. On this perspective, this study seems to fill a gap in the body of knowledge concerning QoL in cancer patients on HPN.

The key message of our study is that HPN is associated with an improvement of QoL in advanced cancer patients. Obviously, the magnitude of this effect may depend on the clinical conditions of the patient requiring a total or a supplemental HPN and on the oncologic stage which can be still amenable or not to further treatments. Thus, we suggest that nutritional support, even in the form of supplemental or total HPN, should not be considered as the last option for incurable cancer patients, but should be properly integrated in a more comprehensive oncologic approach when patients start losing weight or become hypophagic.

## Conflict of Interest

The authors made no disclosures.
